# Population-based prediction of subject-specific prostate deformation for MR-to-ultrasound image registration

**DOI:** 10.1016/j.media.2015.10.006

**Published:** 2015-12

**Authors:** Yipeng Hu, Eli Gibson, Hashim Uddin Ahmed, Caroline M. Moore, Mark Emberton, Dean C. Barratt

**Affiliations:** a Centre for Medical Image Computing, University College London, London, UK; b Diagnostic Image Analysis group, Radboud University Medical Centre, Nijmegen, The Netherlands; c Division of Surgery and Interventional Science, University College London, London, UK

**Keywords:** Statistical shape modelling, Organ motion, Tissue deformation, Kernel regression, Image registration

## Abstract

Statistical shape models of soft-tissue organ motion provide a useful means of imposing physical constraints on the displacements allowed during non-rigid image registration, and can be especially useful when registering sparse and/or noisy image data. In this paper, we describe a method for generating a subject-specific statistical shape model that captures prostate deformation for a new subject given independent population data on organ shape and deformation obtained from magnetic resonance (MR) images and biomechanical modelling of tissue deformation due to transrectal ultrasound (TRUS) probe pressure. The characteristics of the models generated using this method are compared with corresponding models based on training data generated directly from subject-specific biomechanical simulations using a leave-one-out cross validation. The accuracy of registering MR and TRUS images of the prostate using the new prostate models was then estimated and compared with published results obtained in our earlier research. No statistically significant difference was found between the specificity and generalisation ability of prostate shape models generated using the two approaches. Furthermore, no statistically significant difference was found between the landmark-based target registration errors (TREs) following registration using different models, with a median (95th percentile) TRE of 2.40 (6.19) mm versus 2.42 (7.15) mm using models generated with the new method versus a model built directly from patient-specific biomechanical simulation data, respectively (*N* = 800; 8 patient datasets; 100 registrations per patient). We conclude that the proposed method provides a computationally efficient and clinically practical alternative to existing complex methods for modelling and predicting subject-specific prostate deformation, such as biomechanical simulations, *for new subjects*. The method may also prove useful for generating shape models for other organs, for example, where only limited shape training data from dynamic imaging is available.

## Highlights

• A novel framework for building population-predicted, subject-specific models of organ motion is presented.
• Subject-specific PDFs are modelled without requiring knowledge of motion correspondence between training subjects.
• A simple yet generalisable kernel regression scheme is employed.
• A rigorous validation is presented using prostate MR-TRUS image registration data acquired on human patients.

## Graphical abstract

**Figure:**
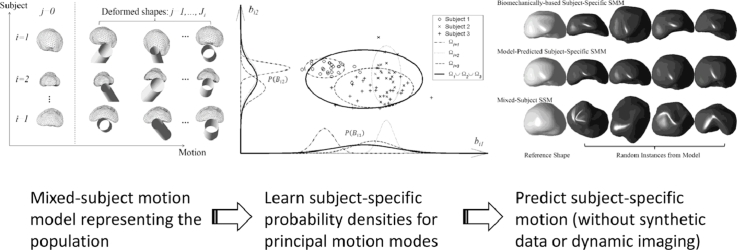

## 1 Introduction

Statistical shape models (SSMs) of soft-tissue organ motion provide a useful means of imposing physical constraints on the displacements allowed during non-rigid image registration, which is especially useful when registering sparse and/or noisy image data (Hawkes et al., 2005, Heimann and Meinzer, 2009). We have used this approach successfully in previous work to compensate for prostate deformation due to transrectal ultrasound- (TRUS-) probe pressure when registering MR and 3D TRUS images of the prostate in the context of MRI-tumour-targeted biopsy and minimally-invasive surgical interventions (Hu et al., 2012, Hu et al., 2011). A growing body of research has investigated a number of alternative solutions to the problem of non-rigid MR-TRUS registration of the prostate, including (semi-) manual approaches (Kuru et al., 2012, Xu et al., 2008), intensity-based approaches (Mitra et al., 2012, Sun et al., 2013) and surface (feature)-based approaches (Narayanan et al., 2009, Sparks et al., 2013, Van de Ven et al., 2015), which are commonly employed in commercial image guidance systems (Marks et al., 2013).

In our approach, a 3D finite element model (FEM) of the prostate is constructed from a segmented T2-weighted MRI scan and biomechanical simulations of possible TRUS-probe-induced gland deformations are used to generate *subject-specific* shape training data for an SSM that represents the likely variation in prostate shape that could occur during a TRUS-guided procedure. The resulting SSM adopts physically realistic shapes and because the model is highly constrained, it can be fitted robustly to sparse and noisy organ surface data (in this case extracted from a 3D TRUS image). Once fitted, the SSM predicts the displacement of all internal points, thus providing a full 3D displacement field within the organ of interest that can be applied to deform the original MR image and, in particular, determine the location of MR-visible lesions within the TRUS volume that are then targeted during biopsy or treatment. Information on the size, shape and location of a target lesion/tumour, as well as additional information, such as the location of vulnerable structures or surgical margins, both of which are important for treatment applications, can be embedded very naturally within such models by labelling the elements within the FEM.

The approach outlined above provides a versatile means of capturing patient-specific data on organ motion, pathology, and anatomy, and data for surgical planning for a wide range of image-guided surgery applications. Physical and statistical models have been combined previously, for example, for simulating spatial image deformations to generate ground-truth data for validating segmentation algorithms (Hamarneh et al., 2008) and for image registration (Wang and Staib, 2000). In the context of our approach, the limitations of using an FEM directly to predict tissue motion are overcome by applying a statistical approach to handle uncertainty in boundary conditions (for example, due to different TRUS probe positions and orientations) and unknown tissue material properties. The need to estimate these parameters in advance is therefore avoided. Instead, multiple biomechanical simulations are performed, each with different combinations of parameter values drawn from physically plausible range. However, simulating subject-specific organ motion using biomechanical modelling efficiently requires specialised software, hardware (such as graphical processing units (GPUs)), and expertise. It is also technically demanding and the need to perform many thousands of simulations for each individual subject becomes computationally expensive, and generating an SSM can take hours in practice even if the degree of manual interaction required can be minimised relatively straightforwardly through the implementation of an automated pipeline process. Furthermore, although there has been considerable methodological progress to ensure the numerical stability of FEM methods, it is widely recognised that such methods can fail to converge under some circumstances, for example due to a poorly configured geometric mesh. Consequently, although integrating such technology into existing clinical workflows is not unsurmountable, there remain a number of significant practical challenges. For this reason, more convenient, computationally efficient, and numerically stable methods for generating subject-specific SSMs of organ deformation – or training data for building them – are highly desirable from the point of view of facilitating clinical adoption.

To date, the popular method described by Cootes et al. (1995) for generating low-dimensional, linear SSMs by applying principal component analysis (PCA) to shape or image training data has been used mainly to generate models that describe organ shape variation across a population, e.g. (Onofrey et al., 2013, Perperidis et al., 2005, Thompson et al., 2008). PCA and other statistical techniques have also been applied to generate subject-specific 4D statistical models for organs undergoing respiratory motion (McClelland et al., 2013) or cardiac motion. Examples include models of the lungs (He et al., 2010, King et al., 2012), the liver (Preiswerk et al., 2014), and the heart (Perperidis et al., 2005). Given the considerable effort required to build a model of organ motion for an individual subject, a number of researchers have investigated so-called population-based or cross-population models (McClelland et al., 2013, Preiswerk et al., 2014). These enable subject-specific organ motion to be predicted using learnt information from an independent training set. It is possible to build a population-based SSM by combining training data that is subject to both inter- and intra-subject organ shape variation, but such models are likely to perform less effectively or efficiently compared with a subject-specific SSM for approximating subject-specific shape/motion. In particular, such models usually require additional constraints, such as that provided by an elastic model (Wang and Staib, 2000), to prevent unrealistic or ‘over-generalised’ instantiation of the model because of shape variation learnt from other subjects.

Multilinear analysis (Vasilescu and Terzopoulos, 2003) has been proposed as a method for dynamic modelling of the heart (Zhu et al., 2008) and cardiac valve (Grbic et al., 2012) motion. Importantly, this approach enables shape variations due to *both* geometric differences between the organs of different subjects (due to anatomical variation) and physiological (or externally-induced) organ motion to be represented by the same statistical model. However, like many related methods in the literature, this method requires known inter-subject motion correspondence; in other words, organ shapes for different subjects must be correlated via an independent signal, such as an ECG. This is very difficult to establish for organs other than the heart and lungs where a physiological signal related to motion is not available or is very difficult to measure. Furthermore, the cardiac models described in Grbic et al. (2012), Zhu et al. (2008) have demonstrated only the ability to predict organ shape at relatively few timepoints given the dynamic data available over the remainder of the cardiac cycle. Klinder et al. (2010) developed a statistical model of motion based on a training set of 4D CT images for 10 patients and used multivariate linear regression to predict lung using the tracked diaphragm motion. In the remainder of this paper, we distinguish between motion (or temporal) correspondence and point correspondence, where motion correspondence refers to linking different shapes of a deforming organ by means of a common timepoint or physiological event.

In this paper, an alternative organ motion modelling method is proposed that is particularly suited to applications such as modelling prostate deformation where a surrogate motion signal (such as a respiratory or cardiac signal) does not exist to establish temporal correspondence between different subjects; the proposed method enables a subject-specific SSM that describes shape variation due to motion to be built without knowing the motion correspondence between subject subspaces. It also requires only limited subject-specific geometric data – for example, a reference shape based on the segmentation of a single (static) MR image – to predict the organ motion for a new (i.e. unseen) subject. The method is also potentially very useful when subject-specific shape training data is too expensive or practically difficult to obtain on each new subject. In this case, the proposed population-based model provides a means of predicting subject-specific motion with the only requirement being a single reference shape that specifies one instance of the shape of the subject's organ. We demonstrate the application of this method for non-rigid registration of MR and TRUS images of the prostate. For convenience, in the remainder of this paper, models that represent physical organ motion are termed *statistical motion models* (SMMs) (Ehrhardt et al., 2011, Hu et al., 2011) to distinguish them from the more general SSM and statistical deformation models (SDMs) where PCA is performed on an image deformation field (Onofrey et al., 2013, Perperidis et al., 2005, Rueckert et al., 2003). SMMs may therefore be considered to be a subset of SSMs.

## 2 Methods

### 2.1 Overview

The underlying concept of the proposed method is that variations in organ shape due to motion can be expressed with respect to a ‘mixed-subject’ – i.e. population-based – SSM that is built using training data from multiple subjects and multiple shapes for each subject. The resulting SSM captures shape variation both *between* and *within* individuals. Kernel regression analysis provides a powerful method for expressing the multivariate subject-specific probability density function (SSPDF), which represents the distribution of *shape parameters* (also known as component scores or weights) related to intra-subject organ motion, as a function of the parameters of a pre-chosen *reference shape*. Once this relationship has been established, the SSPDF that describes the expected organ motion for a new (i.e. unseen) subject can be estimated from new reference shape data for that particular subject. The resulting SSPDF can then be used to construct a subject-specific SMM for the new subject.

**Fig. 1:**
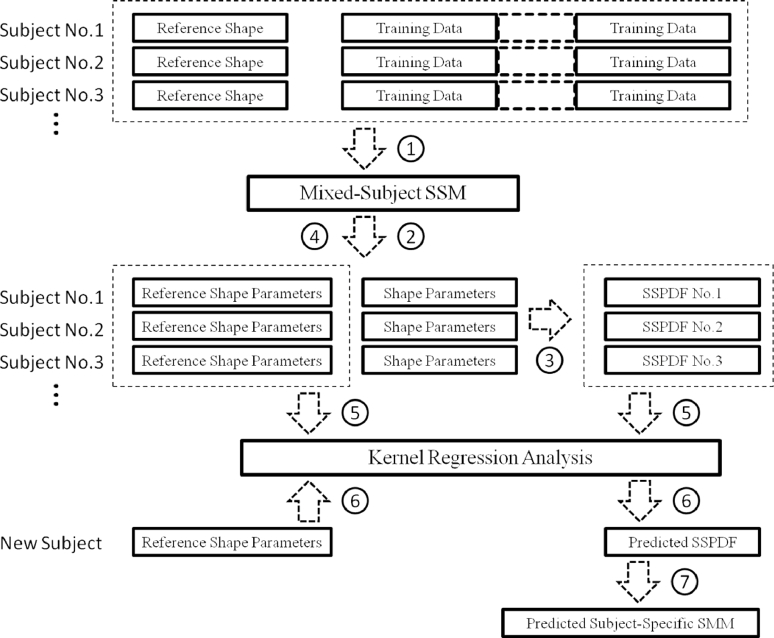
A schematic overview of the proposed method to build a subject-specific SMM.

A schematic overview of the method used to build a subject-specific SMM is shown in Fig. 1. The steps involved are as follows:

1. Build a mixed-subject SSM using all available training data;
2. Obtain the shape parameters for each training dataset with respect to the mixed-subject SSM (e.g. by projection for the case of a linear model);
3. Estimate the SSPDF for each set of shape parameters corresponding to the different training shapes for each subject. The SSPDF may itself be expressed in parametric form and represented by a number of *distribution parameters* (e.g. the mean and variance of a Gaussian distribution);
4. Identify a reference shape for each subject. For example, the reference shape may describe an organ in its ‘resting’, or un-deformed state, or in general at a time corresponding to a particular physiological event. The reference shape is then represented by its shape parameters;
5. Perform kernel regression analysis between the parameters that characterise each SSPDF and the shape parameters that specify the reference shape;
6. Given the reference shape for a new (unseen) subject, calculate the SSPDF for the new subject using regression analysis;
7. Finally, construct a subject-specific SMM for the new subject by using the predicted SSPDF.

The resulting subject-specific SMM is an alternative to a subject-specific SMM built directly from training data available for this subject (including image-based and simulated training data). Therefore, the subject-specific SMM estimated using this method can be compared directly with one generated using the conventional method. In the following sections, an illustration of implementing these steps is provided using the example of building a subject-specific SMM of the prostate that captures deformation caused by the placement of a TRUS probe in the rectum.

### 2.2 Construction of a mixed-subject statistical shape model

**Fig. 2:**
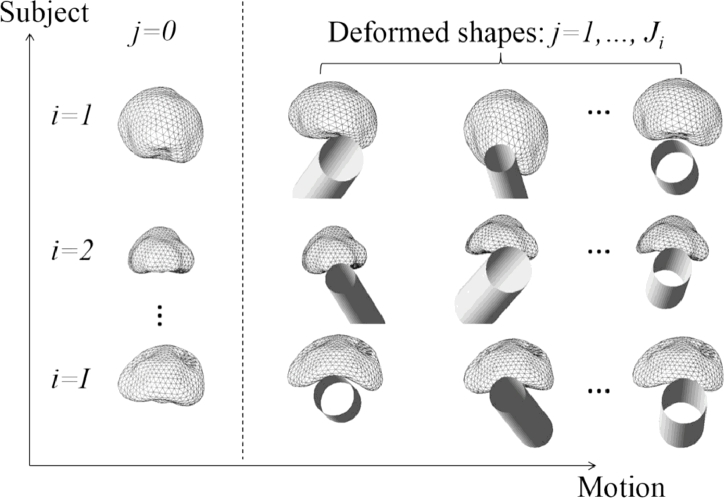
An illustration of deformed prostate shapes for *I* subjects. A reference shape for each subject is denoted by *j* = 0. The 3D position and orientation of the TRUS balloon is represented by a shaded hollow cylinder for each deformed shape instance.

Fig. 2 shows a schematic of the shapes of the prostates of *I* subjects, each represented by triangulated mesh. The shape of each mesh has been simulated using FEM to predict the new deformed shape resulting from the physical deformation of a reference shape. Without assuming an equal number of shapes per subject, varying the pose of the TRUS probe and the diameter of the water-filled balloon surrounding the probe in each simulation results in $J_{i}\;\left(i=1,2,\ldots ,I\right)$ predicted deformed shapes. As described in Hu et al., 2012, Hu et al., 2011), other unknown parameters, such as tissue elastic properties, may also be included as variables in the simulations to reflect uncertainty in these properties. For each subject, the first shape, denoted by j=0, is the reference shape and the remaining $j\;(j=1,2,\ldots ,J_{i})$ shapes are deformed instances of the reference shape. In this example, the *reference shape* represents the prostate in the “resting state”, obtained by segmenting a T2-weighted MR image that was acquired without an endorectal coil (or any other rectal insertion) in place (Hu et al., 2012).

Group-wise surface registration of the meshes can be performed so that: (i) point correspondence between each deformed shape and the reference shape is established for each subject, and (ii) the point correspondence between the reference shapes of different subjects is established. Where FE simulations are performed to synthesise the training dataset, the point correspondence between each deformed shape is known implicitly. Details of the algorithm used in this study to determine cross-patient point correspondence are given in Section 2.7. Once the correspondences are established, the training shapes can be iteratively rigid-aligned to the mean shape. This ensures that intra- and inter-subject variances, such as shapes and sizes, are both preserved.

The mixed-subject SSM can be constructed by applying PCA to $G=I+\sum_{i=1}^{I}J_{i}$ training shape vectors, $s_{g}=[x_{g1},y_{g1},z_{g1},x_{g2},y_{g2},{z_{g2},\ldots ,x_{gN},y_{gN},z_{gN}]}^{T}$, g=1,2,…,G, which each contain the 3D co-ordinates of *N* points that describe the *g*th shape. The shape vectors may define either a 3D surface or a volume, for example, represented by the nodes (vertices) of an FE mesh. Taking advantage of dimensionality reduction by excluding components that explain less variance in the training data, the resulting shape model is approximated by the linear equation using *L* ≤ *G* principal components (Cootes et al., 1995):

(1) $$\begin{array}{ccc} s_{g} & = & \bar{s}+\sum_{l=1}^{L}b_{gl}e_{l}=\bar{s}+\left[e_{1},e_{2},\ldots ,e_{L}\right]{\left[b_{g1},b_{g2},\ldots ,b_{gL}\right]}^{T}\\ & = & \bar{s}+Eb_{g}\; \end{array}$$

where $\bar{s}$ is the mean shape vector; **e**_*l*_ is the eigenvector of the covariance matrix of the (mean-subtracted) training shape vectors corresponding to the *l*th largest eigenvalue,$\;\sigma_{l}^{2}$; and *b_gl_* is a scalar shape parameter; the vector **b**_*g*_ contains the shape parameters that collectively describe the *g*th organ shape. Eq. (1) models mixed-subject individual and motion variations learned from all the training data. An SSM generated in this way is referred to hereon in as a *mixed-subject SSM*.

### 2.3 Subject-specific PDF calculation

The subject-specific probability density for the *i*th subject is denoted by $P(B_{i}:B_{i}\in \Omega_{i})$, where $B_{i}$ is a multivariate random variable of the vector shape parameters and *Ω_i_* ∈ ℜ^*L*^ denotes the *i*th subject subspace. Rearranging (1) we have:

(2) $$\begin{array}{c} b_{ij}=E^{T}\left(s_{ij}-\bar{s}\right) \end{array}$$

In (2) **b***_ij_* contains the shape parameters of the training data by projecting the coordinates **s**_*ij*_ for the *j*th shape belonging to the *i*th subject. Both **s**_*ij*_ and **s**_*g*_ are training shape vectors with different subscripts that denote differently grouped data.

**Fig. 3:**
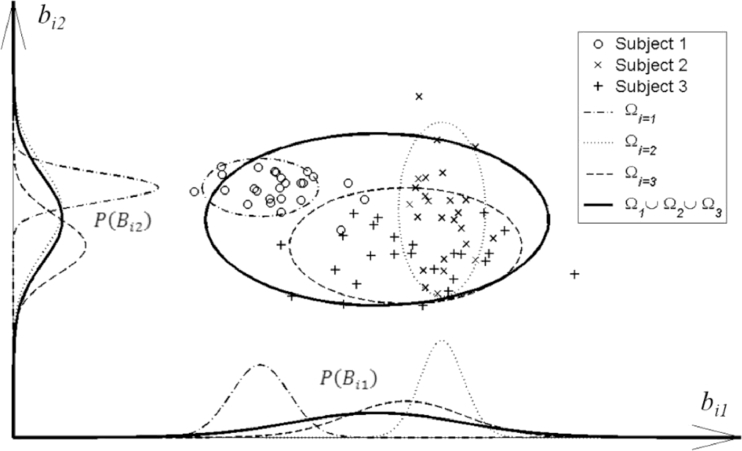
A graphical representation of the factorised probability density *P*(*B_il_*) for three different subjects, each represented by a dashed ellipse containing different data points labelled ○, ×, and +, and for two principal components of the mixed-subject SSM. The curves shown on each axis represent the factorised probability densities, whereas the ellipses represent confidence regions of the SSPDFs, $P(B_{i})$ (see text for details).

$P(B_{i})$ may be simplified by the independence approximation^1^ wherein this multivariate probability density is approximated as a factorised joint probability density, i.e., $P\left(B_{i}\right)≅\prod_{l=1}^{L}P\left(B_{il}\right)$, where $B_{i}={\left[B_{il}\right]}_{l=1,2,\ldots ,L}^{T}$. This has the effect of excluding information on correlation between shape parameters. Expressing the probability in this way enables us to draw an informative plot of the distribution in terms of individual distributions of the scalar random variable *B_il_* for the *l*th shape parameter (corresponding to the *l*th principal component). An example is shown in Fig. 3. The scalar shape parameters $b_{ijl},j=1,2,\ldots ,J_{i}$ are *J_i_* samples of the random variable, *B_il_*.

**Fig. 4:**
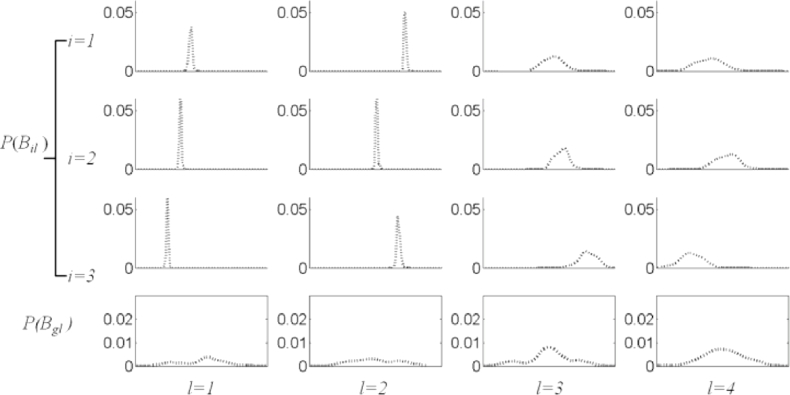
Examples of estimated factorised probability densities *P*(*B_il_*), represented by histograms for the prostate shape data (see text). Each column (from left to right), corresponds to each of the first four principal components (*l* = 1, 2, 3, 4) of the mixed-subject SSM. The first three rows from top represent the first three subjects (*i* = 1, 2, 3). The bottom row represents the population probability densities  *P*(*B_gl_*) computed over the entire training data.

Similarly, the probability density of all the training data that builds the mixed-subject SSM is denoted by $P(B_{g}:B_{g}\in \Omega_{g})$, where the reference space *Ω_g_* is the union of all the subject subspaces. This can be factorised in the same way such that $P\left(B_{g}\right)=\prod_{l=1}^{L}P\left(B_{gl}\right)$. Fig. 4 shows some examples of these factorised probability densities using the histograms of the samples {**b**_*ij*_} from the prostate shape data.

By inspection of the plots in Fig. 4, the following two observations can be made immediately: First, *P*(*B_il_*) is different between subjects and is different from *P*(*B_gl_*) corresponding to the mixed-subject SSM. This provides a potential means to decompose the whole mixed-subject space into motion- and subject subspaces by modelling the SSPDFs. Second, all of the sample distributions have a consistently bell-like shape with different widths and centre positions. Following the independence approximation, the SSPDF may be parameterised by a multivariate Gaussian PDF^1^$N(B_{i};\mu_{i},\mathrm{diag}\left(\sigma_{i}^{2}\right))$, where the distribution parameters, ***μ***_*i*_ and $\mathrm{diag}(\sigma_{i}^{2})$, represent the mean vector and the *L* × *L* diagonal covariance matrix, in which the diagonal entries are the component variance vector $\sigma_{i}^{2}={\left[\sigma_{\mathrm{il}}^{2}\right]}_{l=1,2,\cdots ,L}^{T}$, respectively. This PDF is considered as a parametric example of the SSPDF for *i*th subject, and is entirely characterised by the distribution parameters ***μ***_*i*_ and $\sigma_{i}^{2}$.

### 2.4 Parameter estimation using kernel regression analysis

The distribution parameters may be estimated given a set of samples, $\left\{b_{ij},j=1,2,\ldots ,J_{i}\right\}$. The corresponding maximum likelihood estimators are then given by:

(3) $$\begin{array}{c} \begin{array}{c} {\hat{\mu }}_{i}=\frac{1}{J_{i}}\sum_{j=1}^{J_{i}}b_{\mathrm{ij}} \end{array} \end{array}$$

and

(4) $$\begin{array}{c} \begin{array}{c} {\hat{\sigma }}_{i}^{2}=\frac{1}{J_{i}-1}\sum_{j=1}^{J_{i}}{\left(b_{\mathrm{ij}}-{\hat{\mu }}_{i}\right)}^{2} \end{array} \end{array}$$

Without loss of generality, we now assume that a (nonlinear) relationship exists between the distribution parameter $\theta_{i}={\left[{\hat{\mu }}_{i}^{T},{\hat{\sigma }}_{i}^{T}\right]}^{T}$ of the SSPDF $P(B_{i};\theta_{i})$ and the shape parameters of reference shape **b**_*i*0_ for *i*th subject so that the distribution parameter ***θ***_*i*_, and therefore the SSPDF $P(B_{i})$, may be predicted solely from the shape parameters of the unseen reference shape for a new subject data. In the current study, the distribution parameter is expressed as a linear function of kernels as follows:

(5) $$\begin{array}{ccc} \theta_{m}\left(b\right) & = & \beta_{m0}+\sum_{i=1}^{I}\beta_{mi}K\left(\;b,b_{i0}\right)+\epsilon_{m}\\ & & \mathrm{with}\;\mathrm{the}\;\mathrm{constraint}\;\sum_{i=1}^{I}{|\beta_{mi}|}^{2}\leq c \end{array}$$

In Eq. (5), $K\left(x,\;x^{\prime }\right)=\exp\left(-{∥x-x^{\prime }∥}^{2}/2h^{2}\right)$ is a Gaussian kernel function with kernel parameter *h*, which is determined by a cross validation method described in the Section 2.6. The choice of the kernel function form is briefly discussed in Section 4; *c* is a positive scalar constant; ϵ is a random noise term with its statistical expectation E[ϵ]=0; *m* is the index of each scalar distribution parameter such that $\theta_{i}={\left[\theta_{\mathrm{mi}}\right]}_{m=1,2,\cdots ,2L}$; and $\beta_{m}={\left[\beta_{\mathrm{mi}}\right]}_{i=0,1,\cdots ,I}^{T}$ is a vector *regression parameter*. The optimal regression parameter may be estimated by using a linear least squares technique to minimise the regularised residual sum-of-squares as follows (Hastie et al., 2009): First, a regularised estimator ${\hat{\beta }}_{m}={\left[{\hat{\beta }}_{\mathrm{mi}}\right]}_{i=1,2,\cdots ,I}^{T}$ is given by:

(6) $$\begin{array}{c} \begin{array}{c} {\hat{\beta }}_{m}={\left(\Phi_{m}^{T}\Phi_{m}+\lambda I\right)}^{-1}\Phi_{m}^{T}\theta_{i} \end{array} \end{array}$$

where the design matrix takes the following form:

(7) $$\begin{array}{c} \Phi_{m}=\left[\begin{array}{ccc} K\left(b_{10},b_{10}\right)-{\bar{\varphi }}_{1} & \cdots & K\left(b_{10},b_{I0}\right)-{\bar{\varphi }}_{I}\\ \vdots & \ddots & \vdots \\ K\left(b_{I0},b_{10}\right)-{\bar{\varphi }}_{1} & \cdots & K\left(b_{I0},b_{I0}\right)-{\bar{\varphi }}_{I} \end{array}\right] \end{array}$$

${\bar{\varphi }}_{k}=\frac{1}{I}\sum_{i=1}^{I}K\left(\;b_{k0},b_{i0}\right)$, **I** is the identity matrix, and *λ* is the ridge weighting parameter. In practice, the regularisation parameter *λ* is set to a small constant to avoid over-fitting while maintaining acceptable residuals; $\lambda =10^{-8}$ was used in all the experiments presented in this study. The offset coefficient is then given by:

(8) $$\begin{array}{c} {\hat{\beta }}_{m0}=\frac{1}{I}\sum_{i=1}^{I}\theta_{mi}-\sum_{k=1}^{I}{\hat{\beta }}_{mk}{\bar{\varphi }}_{k} \end{array}$$

### 2.5 Prediction of a subject-specific SMM

Given reference shape data for a new subject, the shape parameters **b**_new, 0_ for the new subject can be estimated by first non-rigidly registering to the mean shape of the group-wise registration (see details in Section 2.7), and then projecting onto the principal components of the mixed-subject SSM after removing the rigid component. Thus,

(9) $$\begin{array}{c} b_{\mathrm{new},0}=E^{T}\left(s_{\mathrm{new},0}-\bar{s}\right) \end{array}$$

where **s**_new, 0_ is the rigidly-aligned, undeformed shape. Each distribution parameter of a new SSPDF can then be computed by taking the conditional expectation of Eq. (5), as follows:

(10) $$\begin{array}{c} \theta_{m}\left(b_{\mathrm{new},0}\right)=E\left[\Theta |\;b_{\mathrm{new},0}\right]=\beta_{m0}+\sum_{i=1}^{I}\beta_{mi}K\left(\;b_{\mathrm{new},0},b_{i0}\right) \end{array}$$

where coefficients $\beta_{mi}^{\mathrm{new}}$ are given by Eqs. (6) and (8). The SSPDF $P(B_{\mathrm{new}}:B_{\mathrm{new}}\in \Omega_{\mathrm{new}})$ for the new subject can now be predicted using the predicted distribution parameters, $N(B_{\mathrm{new}};\mu_{\mathrm{new}},\mathrm{diag}\left(\sigma_{\mathrm{new}}^{2}\right))$.

Once $P(B_{\mathrm{new}})$ has been estimated, the linear model may be obtained directly by “centering” the predicted diagonal covariance matrix, so that the predicted subject-specific SMM takes the form:

(11) $$\begin{array}{c} \begin{array}{c} s_{\mathrm{predict}}=\bar{s}+{E\mu }_{\mathrm{new}}+Eb_{\mathrm{new}} \end{array} \end{array}$$

where the new component variance becomes $\sigma_{\mathrm{new}}^{2}$, $\bar{s}+{E\mu }_{\mathrm{new}}$ is equivalent to the mean of the predicted subject-specific SMM and **b**_new_ represents the new shape parameters.

### 2.6 Optimal kernel parameter

For each regression kernel parameter, expressed as $h=10^{x}$, an optimal value is computed by minimising the cross validation error, defined as the root-mean-square of the regression residuals, as in Eq. (5). The regression error is computed for each data in a leave-one-out scheme by comparing the difference between the ground-truth distribution parameters, computed from the training data via Eqs. (3) and (4), and the predicted distribution parameters, computed from the test data via Eqs. (6), (8) and (10). In this study, a golden search strategy was used to then find the optimal value of *x* within the predefined interval 1 ≤ *x* ≤ 8, with the cross validation error serving as the objective function to minimise.

### 2.7 Point correspondence

One of the advantages of the proposed modelling technique is that it does not require the establishment of motion correspondence between the subject subspaces for different subjects (also described in Section 1) since only the probability densities are modelled to describe the subject motions, motion data can be grouped in an arbitrary order in the training dataset, which overcomes a number of practical difficulties. However, point correspondence still needs to be established between subject subspaces and may be estimated using, for example, a group-wise surface registration scheme (Heimann and Meinzer, 2009).

In this study, inter-subject registration of the training shapes required to build the mixed-subject SSM was performed using an iterative group-wise registration scheme based on the landmark-guided coherent point drift (LGCPD) method (see Hu et al., 2010a for more details), with anatomical apex and base points of the prostate gland serving as two known correspondent points to assist the registration in finding the *point correspondence* between organ surfaces. In this scheme, the mean shape of the registered segmentations was updated iteratively until convergence. Typically, this took no more than five iterations. Because each deformed shape was generated by using an FEM simulation to predict a physical deformation of the reference shape, with the final deformed shape represented by a 3D FE mesh, the 3D point correspondence between different deformed shapes for each subject is known from matching the corresponding nodes (vertices) in the reference and deformed meshes. Finally, a single pair-wise registration using the same method was performed to find point correspondence between a new reference shape for an unseen subject and the mean shape found following the group-wise registration.

### 2.8 Validation methodology

#### 2.8.1 Data acquisition

To test the method introduced in the previous sections for a real-world application, a subject-specific SMM of an unseen prostate gland was built and compared with an SMM generated directly using biomechanical modelling using the methods described in detail in Hu et al. (2012). The mixed-subject SSM was built using 100 FEM simulations of TRUS-probe-induced gland deformation for each of 36 patients, leading to 3636 training shapes in total. For each simulation, different probe/balloon positions and orientations, different balloon diameters, and different elastic material properties were applied (see Hu et al., 2012 for further details). For each of the 36 patients, the reference geometry of the prostate was defined as the shape resulting from a manual segmentation of the capsule in a T2-weighted MR scan, performed by an expert clinical observer (an experienced radiologist or a urologist with an additional verification of the segmented contours by an experienced radiologist).

#### 2.8.2 Cross validation

**Fig. 5:**
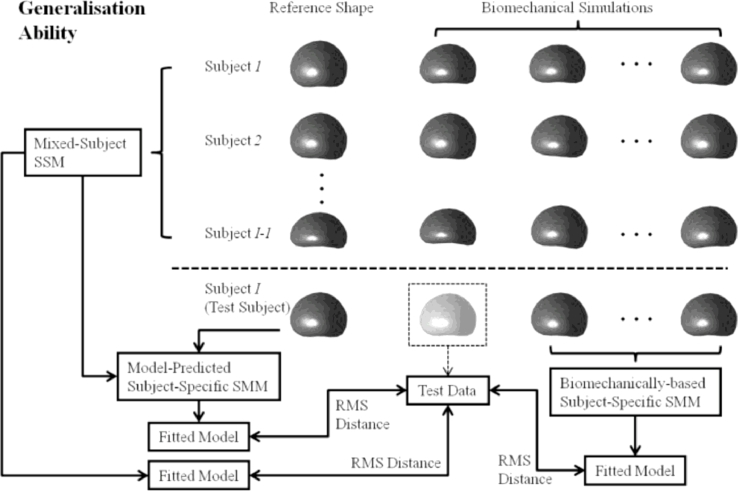
An overview of the leave-one-out methodology used to compare the modelling ability of three linear models by computing the RMS-distance-based generalisation ability. The boxed shape with a lighter shading denotes the test data that is compared to each of the three models in the leave-one-out scheme.

**Fig. 6:**
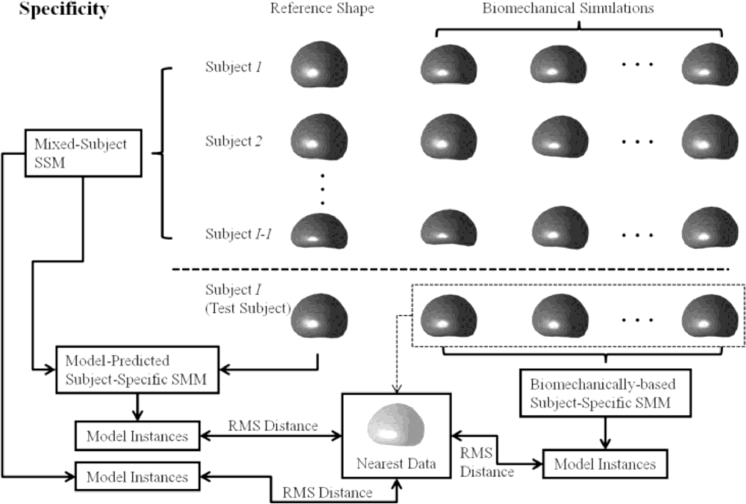
An overview of the leave-one-out methodology used to compare the modelling ability of three linear models by computing the RMS-distance-based specificity.

A leave-one-out, cross-validation framework was used to assess the *generalisation ability* and *specificity* (defined in Hu et al., 2010b, Styner et al., 2003) of the following three linear models: (a) a subject-specific SMM, generated using the population-based model proposed in this paper, (b) a subject-specific SMM based on biomechanical simulation training data and, for comparison, (c) a mixed-subject SSM built using a training dataset that represents both inter- and intra-subject organ shape variation (this model is by definition not subject-specific). Figs. 5 and 6 illustrate the leave-one-out validation method used for a chosen *test subject*. The three linear models are constructed independently. The root-mean-square (RMS)-distance-based generalisation ability and specificity then can be computed for each test subject. The cross validation method described below provides an overall assessment of the modelling ability. Low RMS distances indicate a strong model generalisation ability and specificity.

The generalisation ability of a linear model quantifies its ability to describe unseen data, which relates closely to the application of interest in this paper, namely, capturing organ motion to provide prior information for registering non-rigidly to unseen (TRUS image) data. It was measured by a separate, embedded leave-one-out scheme (Hu et al., 2010b). The generalisation ability was defined as the RMS Euclidean distance between the mesh nodes of an unseen *test data* and the corresponding nodes of the instantiated model fitted to the test data (i.e. the *fitted model*). In this study, the unseen test data (as denoted in boxed prostate shape with a lighter shading in Fig. 5) was the data left out from the 100 biomechanical simulations of the test subject in the embedded leave-one-out scheme; the biomechanically-based SMM was built independently using the remaining 99 simulations, as illustrated in Fig. 5. The RMS-distance-based generalisation ability is given by:

(12) $$\begin{array}{c} \mathrm{RM}S_{\mathrm{gen}}=\sqrt{\frac{1}{N}{\left(s_{\mathrm{test}}-s_{\mathrm{fitted}}\right)}^{T}\left(s_{\mathrm{test}}-s_{\mathrm{fitted}}\right)} \end{array}$$

where *N* is the number of the mesh nodes of each model, **s**_test_ and **s**_fitted_ are the shape vectors (as defined in Section 2.2) of a test data and the instantiated model, respectively. The generalisation abilities were computed for the three linear models in the main “subject-level” leave-one-out scheme.

It is also important to note that, to avoid bias, a different leave-one-out scheme was used to validate the linear models versus the estimation of the optimal kernel parameter described in Section 2.6: In the validation experiments, each of the 36 model-predicted subject-specific SMMs was tested using a mixed-subject SSM generated from the remaining 35 training datasets. Among these, 34 subjects were used as training data to compute the regression error for the remaining datasets in order to determine the optimal kernel parameter for the regression.

The specificity of each linear model was also computed using the same cross-validation framework, which is similar to that adopted in Hu et al. (2010b). This measure indicates the degree to which the deformations of a linear model are constrained, which is relevant because it is desirable for the model to be robust to corrupted data, for instance, due to image artefacts or noise. Furthermore, the model should be able to predict missing data. For the purposes of this study, as illustrated in Fig. 6, this measure was defined as the RMS distance between each of a number of randomly sampled *model shape instances*, specified by **s**_instance_, and the *nearest shape* found in the training data (i.e. 100 biomechanical simulations), specified by **s**_nearest_, as follows:

(13) $$\begin{array}{c} \mathrm{RM}S_{\mathrm{spc}}=\sqrt{\frac{1}{N}{\left(s_{\mathrm{instance}}-s_{\mathrm{nearest}}\right)}^{T}\left(s_{\mathrm{instance}}-s_{\mathrm{nearest}}\right)} \end{array}$$

where *N* is number of solid mesh nodes in the model. For each test subject, one thousand deformed prostate glands for each linear model were generated by randomly sampling **b** from $P(B_{\mathrm{new}})$, $P(B_{i})$ and $\;P(B_{g})$, respectively. The prostate shape instances generated using each linear model form a set that defines the model space, and the distance to the nearest training data from the random instance measures the specificity of the linear model.

For comparison, the generalisation ability and specificity of a set of “*k*-nearest” SSMs were computed for only the *k* nearest training subjects are used, based on the RMS distances between the reference shape of the available training subject and that of the test subject. Therefore, when *k* > 1 the *k*-nearest SSM is a mixed-subject SSM, whereas a single-subject SMM is constructed when *k* = 1.

#### 2.8.3 SMM-based registration validation

Although the main contribution of this paper is the presentation of an alternative technique for generating a subject-specific SMM using synthesised training data, it is also important to assess the ability of such models to recover actual patient organ motion as part of a non-rigid image registration algorithm. To satisfy this, the accuracy of registering a deformable, model-predicted subject-specific SMM, which is based on MR-derived prostate geometry data, to 3D TRUS images was investigated by quantifying the target registration error (TRE) in the alignment manually-identified, independent anatomical landmarks for 8 patient datasets following registration using the method described in our previous published work (Hu et al., 2012). The data for these 8 patients was independent of the training data used to build the predictive model. This TRE provides an independent measure of the registration performance that can be compared directly with registrations that make use of SMMs built using the results of biomechanical simulations of prostate motion for each patient.

## 3 Results

**Fig. 7:**
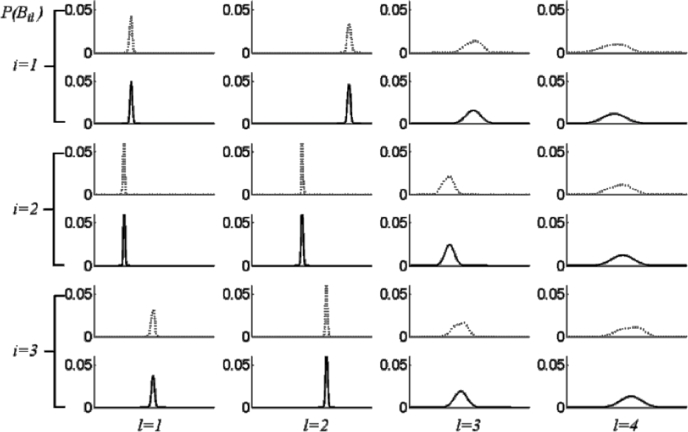
Example plots of the factorised *P*(*B_il_*) for the first four predicted SSPDFs for three new test subjects (solid line), compared with the histogram constructed using original {**b**_*ij*_} (dotted line). It can be seen that the corresponding curves show excellent agreement.

Fig. 7 shows example histograms (plotted as dotted lines) representing *P*(*B_il_*) for the data used in this study, and the regression-estimated subject-specific probability density curves (plotted as solid lines) for first four principal components for three patients. The goodness-of-fit between the corresponding curves was evaluated using the *X*^2^ test.^2^ The result – an average *p* > 0.78 – indicates excellent agreement and provides justification for the effectiveness of the kernel regression analysis and the choice of the Gaussian form to model the PDFs in this study.

**Fig. 8:**
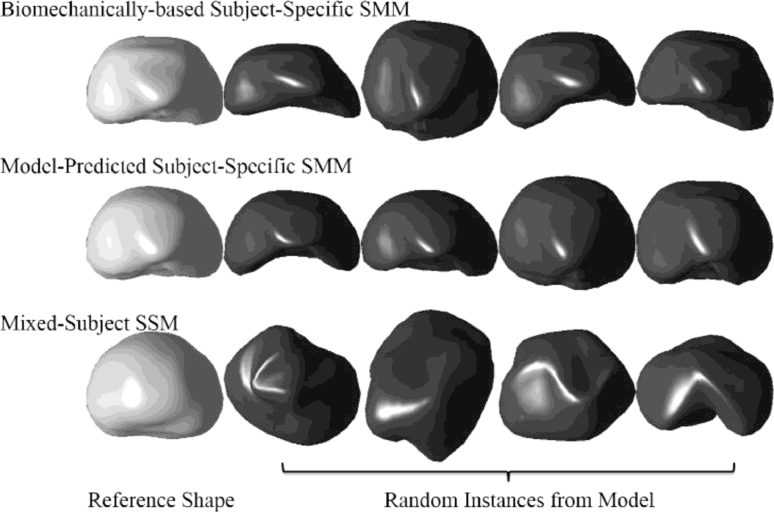
Top row: The randomly sampled prostate glands from the ground-truth biomechanically-based SMM of a test subject (as in the leave-one-out validation). Middle row: Samples from the model-predicted subject-specific SMM, which are constructed from data excluding the test subject. Bottom row: Samples from the mixed subject SSM which includes both intra- and inter-subject shape variations in the training data. The first column shows the reference shape from each model.

Fig. 8 shows examples of random shape instances generated using the biomechanically-based SMM (used here as the ground-truth), the model-predicted subject-specific SMM of a prostate for the same subject, and the mixed-subject SSM (which captures the general shape variation over the training population of 36 patient prostates). By comparing the general form of the shapes generated using the three methods (see Fig. 8), it is visually evident that the subject-specific SMM generates shapes look more physically realistic than those generated by the mixed-subject SSM, and are closer in appearance to those obtained from the ground-truth biomechanically-based SMM. (It should be noted that because the shape instances shown in Fig. 8 are based on random sampling, they are purely illustrative of the form of shapes generated by each SMM, and therefore should be compared group-wise, between rows, and not down each column.)

**Fig. 9:**
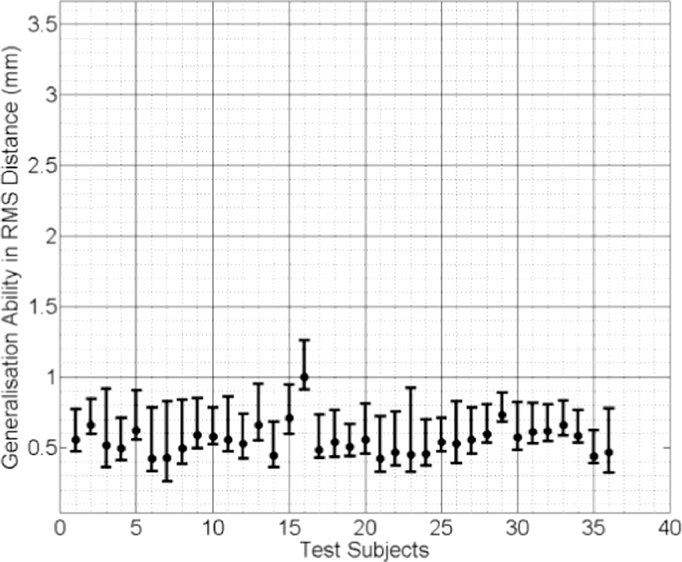
Generalisation ability of the model-predicted subject-specific SMM for each test subject, generated using the proposed method and expressed as the median RMS distance (the error bars indicate the 5th/95th percentiles of these RMS distances).

**Fig. 10:**
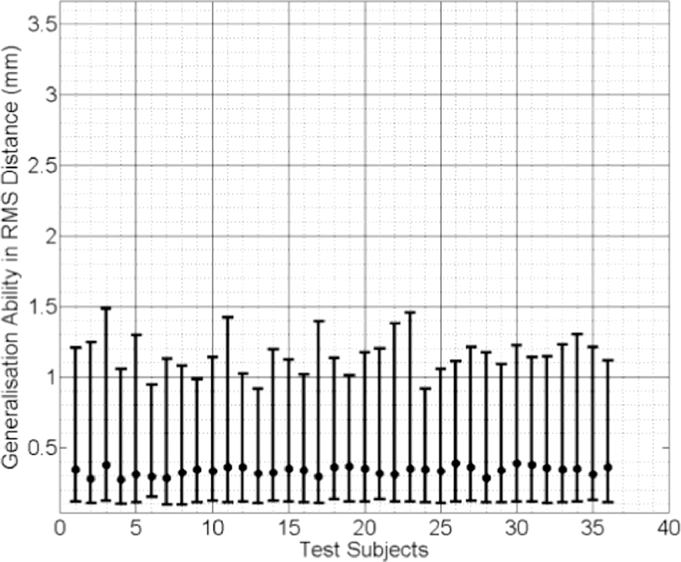
Generalisation ability of the biomechanically-based subject-specific SMM for each test subject, generated using the (ground truth) biomechanical simulations and expressed as the median RMS distance (the error bars indicate the 5th/95th percentiles of these RMS distances).

**Fig. 11:**
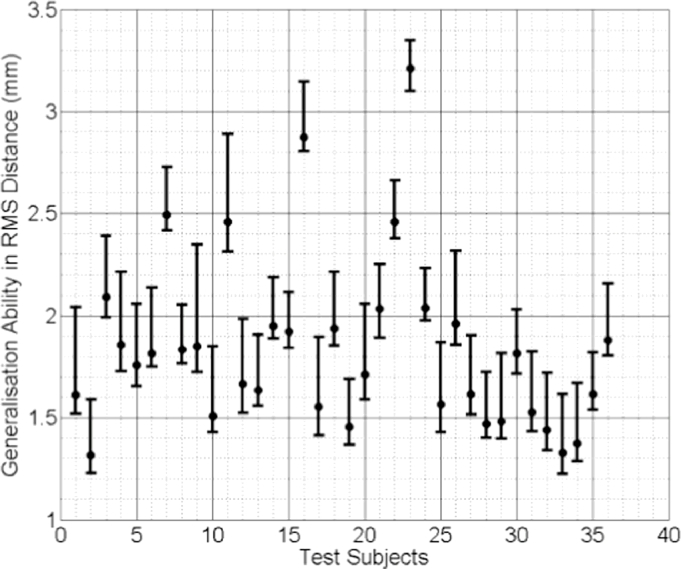
Generalisation ability of the mixed-subject SSM for each test subject, expressed as the median RMS distance (the error bars indicate the 5th/95th percentiles of these RMS distances).

In Figs. 9, 10 and 11 the median RMS value of the generalisation ability of the model-predicted-, biomechanically-based subject-specific SMM and the mixed-subject SSM for each test subject are plotted, respectively. Inspection of these plots reveals that the two subject-specific SMMs provide lower RMS errors compared with the mixed-subject SSM. Using a confidence level of 0.05, paired Kolgomorov–Smirnov tests confirm that: (a) mixed-subject SSM has significantly lower generalisation ability than both the model-predicted- and the biomechanically-based SMM (*p* < 0.0001 in both cases); and (b) the difference in generalisation ability between the model-predicted- and biomechanically-based SMMs is not significantly larger than 0.1 mm (*p* < 0.0001). Therefore, we conclude that the proposed model-predicted SMM has comparable generalisation ability to unseen data to that of the biomechanically-based SMM, while both outperform the mixed-subject SSM in terms of this measure.

**Fig. 12:**
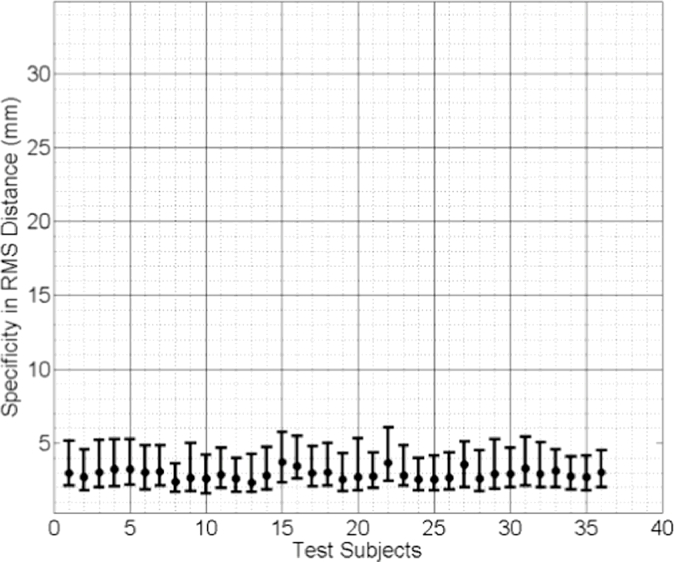
Specificity of the model-predicted subject-specific SMM for each test subject, expressed as the median RMS distance (the error bars indicate the 5th/95th percentiles of these RMS distances).

**Fig. 13:**
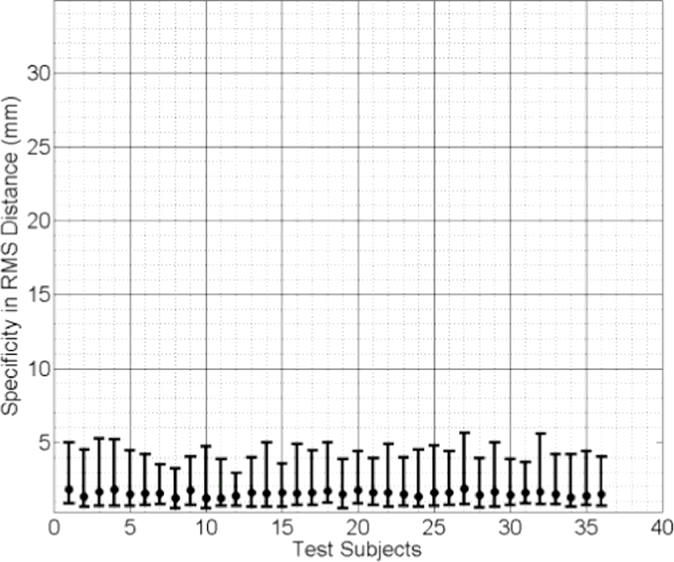
Specificity of the biomechanically-based mixed-subject SSM for each test subject, expressed as the median RMS distance (the error bars indicate the 5th/95th percentiles of these RMS distances).

**Fig. 14:**
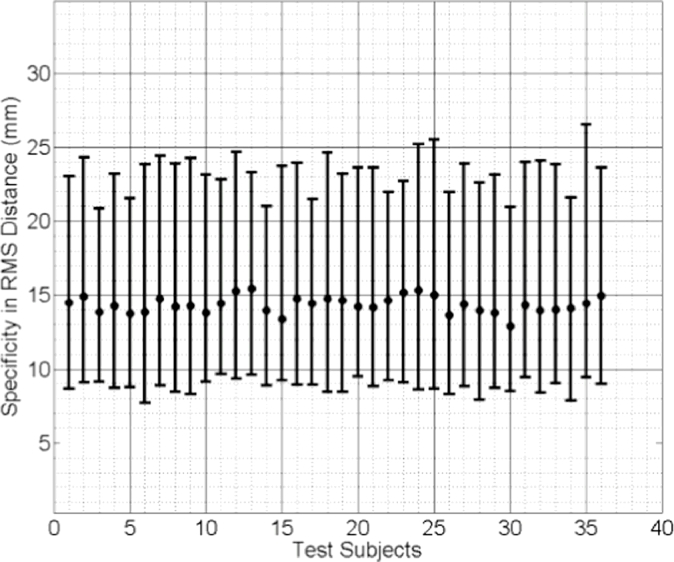
Specificity of the mixed-subject SSM for each test subject, expressed as the median RMS distance (the error bars indicate the 5th/95th percentiles of these RMS distances).

The median values of the specificities of the three linear models are plotted in Fig. 12, Fig. 13, Fig. 14. Comparing these results reveals that the subject-specific SMMs provide significantly smaller (therefore better) model specificities. The same statistical test concludes that the difference in specificity between the mixed-subject SSM and either of the other two subject-specific SMMs is significantly larger than 10 mm, with *p* < 0.0001. However, the difference between the two subject-specific SMMs is not greater than 1 mm (*p* = 0.0005). These results indicate that, compared to the subject-specific SMMs, the ability of the mixed-subject SSM to generate accurate subject-specific data is poor. Furthermore, compared to the biomechanically-based SMM, the proposed model-predicted SMM provides equivalent modelling ability in terms of generating subject-specific instances.

**Fig. 15:**
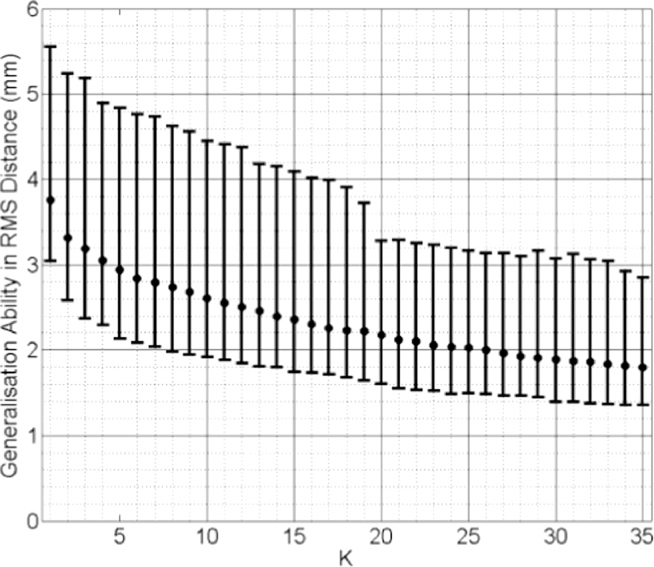
Generalisation ability of the *k*-nearest SSMs plotted versus increasing values of *k*. Pooled data from all test subjects were used, expressed as the pooled median RMS distance (the error bars indicate the 5th/95th percentiles of these RMS distances).

**Fig. 16:**
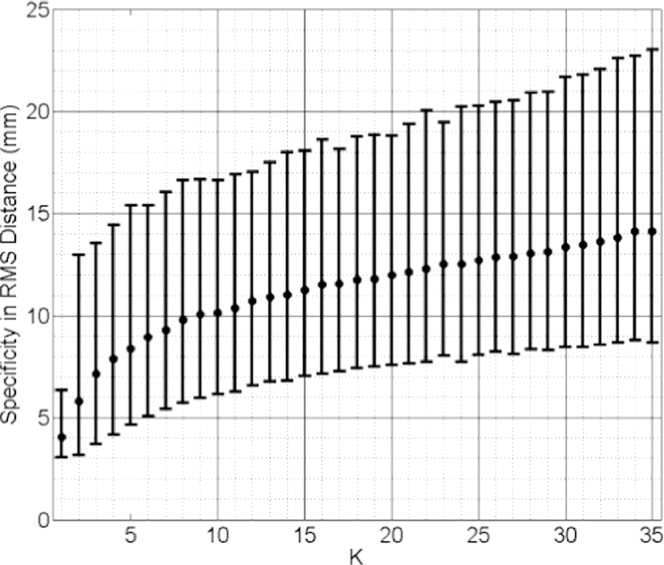
Specificity of the *k*-nearest SSMs plotted versus increasing value of *k*. Pooled data from all test subjects were used, expressed as pooled median RMS distance (the error bars indicate the 5th /95th percentiles of these RMS distances).

Median values of generalisation ability and specificity of the *k*-nearest-SSMs are plotted in Figs. 15 and 16, both calculated using pooled test subjects from the cross validation scheme. Inspecting these results reveals that the generalisation ability increases (RMS distance error decreases) as *k* increases. The best generalisation ability (= 3.76 mm median RMS distance) was achieved when *k* = 35. This distance is close to that of the mixed-subject SSM reported in Fig. 11 and can be improved significantly (*p* < 0.0001) by adopting a model-predicted SMM (Median RMS distance = 0.57 mm; Fig. 9). The specificity, on the other hand, decreases as more training subjects are included: the smallest median RMS distance (4.06 mm) was obtained using only the closest training subject, i.e. *k* = 1, and is significantly worse (*p* < 0.0001) than that calculated for the model-predicted SMM (Median RMS distance = 2.90 mm; Fig. 12).

From the results above, it follows that the generalisation ability of a *k*-nearest-SSM is likely to improve as more training data become available. However, this clearly imposes a practical limitation on this approach and increasing the number of training shapes has the undesirable effect of increasing the model specificity, meaning that shapes instantiated by the model become less physically plausible (as indicated in Fig. 8).

The TRE results using the proposed method for generating subject-specific SMMs are summarised in Table 1, along with published TRE data obtained by registering biomechanically-based subject-specific SMMs (Hu et al., 2012). With a confidence level set to 0.05, a paired Kolmogorov–Smirnov test indicates that there is no significant difference between the TREs obtained using the two methods (*p* = 0.14). This suggests that the proposed method for generating subject-specific SMMs provides an alternative to conventional modelling techniques that require subject-specific training data without compromising registration accuracy.

## 4 Discussion

This paper describes a new framework for modelling subject-specific organ motion in which learnt statistics from a training population are used to *predict* subject-specific training data for an unseen subject rather than requiring those data to be provided directly either from subject-specific dynamic image data or from subject-specific computer simulations, both of which can often place a significant burden on technical and healthcare resources. In particular, the proposed method allows subject-specific organ motion to be modelled implicitly *without knowledge of the explicit motion correspondence between different subjects* (which for respiratory organ motion for example, might be provided by an independent respiratory signal or surrogate respiratory signal). The proposed motion modelling method was compared with biomechanical modelling as an alternative, direct means of generating subject-specific synthetic training data. One advantage of using biomechanical simulations is that the point correspondences between successive shapes of the organ of a particular subject are known implicitly, since these are computed relative to a common reference shape. In general, however, point correspondence may be established via any of a number of point registration methods described in the literature (Heimann and Meinzer, 2009).

Further work is necessary to validate the technique against image-derived organ shape data for a wider variety of applications, but a key potential advantage of the method over alternative approaches is that only limited subject-specific data on motion-related organ shape change are required. This makes the method both computationally efficient and highly suited to applications where more comprehensive data on organ motion, such as a 4D image with a high temporal resolution, are difficult or impossible to acquire. In situations when dynamic imaging of organ motion is feasible, but has significant practical constraints, such as limited temporal resolution or limited access to the required imaging facilities, the proposed method can in principle work with only a small number of training shape instances and therefore may be usefully applied. Moreover, the requirement for a single reference shape for unseen subjects overcomes practical constraints that are commonly encountered in the clinical setting where a segmentation from a (static) diagnostic or planning image is often the only, or at least most readily accessible, data available.

In the example used in this study, subject-specific prostate SMMs were built to describe the motion of the prostate gland alone, but the method could also be extended to model multi-organ motion. Furthermore, the proposed framework may be adapted easily to use a different kernel function, i.e. *K* in Eq. (5), a different regression technique and/or another PDF, such as a mixture model for cases where a multi-modal distribution is observed. The simple Gaussian function form *K* takes in Eq. (5) is proposed mainly for its efficiency in local weighting and prevalence in wider statistical learning applications. This choice is proven adequate in this case based on the cross validation results presented in Section 3, but another kernel function might be equally valid. Although these adaptations would not necessarily result in a direct linear model represented by Eq. (11), random samples of the subject-specific organ shape can be drawn from the learnt SSPDF, for example, using a Monte Carlo approach, which are then used to build a linear SMM using a standard PCA-based or other model construction method.

Reference shapes were included when building the mixed-subject SSM so that these predictors can be expressed using the same SSM. However, this may introduce a small bias into the model. To investigate this further, we calculated reconstruction errors in RMS distance using the mixed-subject SSMs with- and without the reference shape data. These were 0.28 ± 0.065 mm and 0.28 ± 0.065 mm, respectively; no statistical significant difference can be concluded with *p* = 0.58 and a confidence interval on the mean difference of [−0.0028, 0.0016], based on a pooled two sample *t*-test. We therefore conclude that the impact of including the reference shape was negligible. Any other linear form of parameterisation of these predictors should have equivalent performance in the subsequent regression analysis. In theory, other nonlinear parameters representing the reference shape and/or other predictors, such as intra-procedural measurements (e.g. gland size) and temporal information, can readily be incorporated in the proposed learning framework. These may help predict the subject-specific SMM but this hypothesis would need further investigation beyond the scope of the present study.

**Fig. 17:**
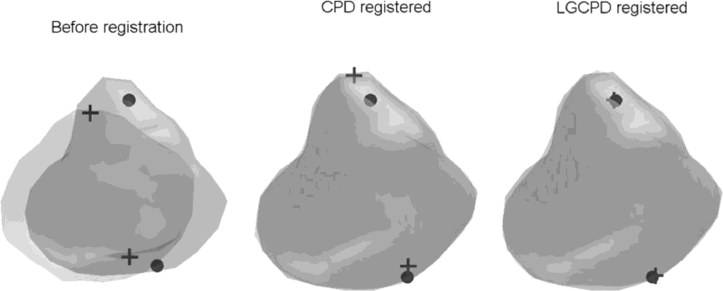
Example of pair-wise registration of prostate surfaces and anatomical landmarks (apex and base) using the CPD and LGCPD algorithms. It can be seen that the landmarks are well aligned (right) after using the LGCPD algorithm, compared with using the CPD algorithm (middle).

A secondary noteworthy aspect of the work is the use of the group- and pair-wise LGCPD algorithms to non-rigidly register training shapes (see Section 2.7). Fig. 17 shows an example of a pair-wise registration of prostate surfaces. This algorithm provides a faster and more robust extension to the general-purpose CPD algorithm, originally proposed by Myronenko and Song (2010).

The value of *L* in Eq. (1) may be chosen so that the reference SSM covers of a certain percentage of the cumulative variance (e.g. at least 99%, yielding *L* = 31 in this study) in the training data. An interesting observation is that the proposed method may be useful for determining an optimal value of *L* as the components ordered with decreasing variance may contain too much noise to be reasonably modelled by a Gaussian distribution or captured by kernel regression. However, further investigation of this point is beyond the topic of this paper and remains to be investigated in future work.

Importantly, for the application of modelling prostate motion to enable non-rigid registration of MR to TRUS images, the proposed method reduces the time required to build a subject-specific SMM substantially, compared with using subject-specific biomechanical simulations to provide model training data. The time taken to generate a subject-specific SMM in this study was on average less than 20 seconds in total (∼18 s for the single LGCPD registration and <2 s for regression evaluation) compared with at least a few hours required for GPU-based FEM simulations (Hu et al., 2012, Hu et al., 2011). This means that model generation is no longer only practical as a pre-operative step within an image-guided surgery workflow, but could feasibly be performed immediately prior to or even during a procedure, which may have significant practical advantages in terms of convenience in the clinical setting. In addition, the proposed model generation method does not require the resources demanded by FE simulation, which is difficult to automate to a level that they can be performed by clinicians without significant technical support or at least in-depth training. Moreover, potential issues regarding numerical instability and lack of convergence are avoided, and high-quality FE simulations need only be limited to generating training data, which in principle only needs to be done once to create a single generative model from which subject-specific SMMs are built.

## Figures and Tables

**Table 1:** Summary of TREs before and after registration using model-predicted versus a biomechanically-based, subject-specific SMMs.

	Subject	1	2	3	4	5	6	7	8	All
Median (95% percentile) TRE (mm)	Start	9.42 (11.39)	14.52 (17.43)	6.29 (9.62)	6.25 (9.42)	9.32 (11.14)	5.86 (8.75)	8.84 (11.65)	6.15 (8.98)	8.13 (15.02)
	Model-predicted, subject-specific SMM	2.88 (7.94)	3.95 (10.75)	1.79 (6.86)	1.98 (4.99)	2.81 (7.16)	1.90 (6.09)	2.79 (9.26)	1.92 (5.65)	2.40 (6.19)
	Biomechanically-based subject-specific SMM	2.68 (7.21)	3.19 (9.62)	1.69 (5.38)	1.56 (5.21)	2.60 (6.84)	1.58 (4.65)	2.92 (7.49)	1.49 (4.66)	2.42 (7.15)

## Footnotes

1 The multivariate Gaussian assumption and the independence approximation have been discussed extensively in the literature (e.g. see Shlens, 2005). We report a maximum correlation of 0.36 for all of the individual subjects, with no significant correlation observed, based on data in this study. It is noteworthy that a more complicated, non-Gaussian distribution, such as a non-unimodal mixture model, or a full covariance matrix, may be considered when necessary (e.g. to describe certain pathological shape variations). The impact of the independence approximation is dependent on the application and here is assessed by the cross validation and image registration accuracy. In practice, this approximation reduce the degrees of freedom of the covariance matrix but maintains the modelling generalisation ability as demonstrated in the cross validation (see Section 3).

2 Unless otherwise indicated, significance levels of all the statistical tests used in this study were set to *α* = 0.05.
